# Azithromycin Mitigates Cisplatin-Induced Lung Oxidative Stress, Inflammation and Necroptosis by Upregulating SIRT1, PPARγ, and Nrf2/HO-1 Signaling

**DOI:** 10.3390/ph16010052

**Published:** 2022-12-29

**Authors:** Emad H. M. Hassanein, Ghadir A. Sayed, Abdullah M. Alzoghaibi, Abdalmohsen S. Alammar, Basel A. Abdel-Wahab, Omnia A. M. Abd El-Ghafar, Somya E. Mahdi, Ahmed M. Atwa, Mohammed A. Alzoghaibi, Ayman M. Mahmoud

**Affiliations:** 1 Department of Pharmacology and Toxicology, Faculty of Pharmacy, Al-Azhar University, Assiut 71524, Egypt; 2 Department of Biochemistry, Faculty of Pharmacy, Egyptian Russian University, Cairo 11829, Egypt; 3 College of Medicine, King Saud University, Riyadh 11461, Saudi Arabia; 4 Dental Department, Hail Health Cluster, Ministry of Health, Hail 55421, Saudi Arabia; 5 Department of Medical Pharmacology, College of Medicine, Assiut University, Assiut 71515, Egypt; 6 Department of Pharmacology, College of Pharmacy, Najran University, Najran 55461, Saudi Arabia; 7 Department of Pharmacology and Toxicology, Faculty of Pharmacy, Nahda University, Beni-Suef 62521, Egypt; 8 Department of Physiology, Faculty of Medicine, Zagazig University, Zagazig 44519, Egypt; 9 Department of Pharmacology and Toxicology, Faculty of Pharmacy, Egyptian Russian University, Cairo 11829, Egypt; 10 Physiology Department, College of Medicine, King Saud University, Riyadh 11461, Saudi Arabia; 11 Department of Life Sciences, Faculty of Science and Engineering, Manchester Metropolitan University, Manchester M1 5GD, UK; 12 Physiology Division, Zoology Department, Faculty of Science, Beni-Suef University, Beni-Suef 62514, Egypt

**Keywords:** chemotherapy, necroptosis, lung injury, oxidative stress, cisplatin, inflammation

## Abstract

Acute lung injury (ALI) is one of the adverse effects of the antineoplastic agent cisplatin (CIS). Oxidative stress, inflammation, and necroptosis are linked to the emergence of lung injury in various disorders. This study evaluated the effect of the macrolide antibiotic azithromycin (AZM) on oxidative stress, inflammatory response, and necroptosis in the lungs of CIS-administered rats, pinpointing the involvement of PPARγ, SIRT1, and Nrf2/HO-1 signaling. The rats received AZM for 10 days and a single dose of CIS on the 7th day. CIS provoked bronchial and alveolar injury along with increased levels of ROS, MDA, NO, MPO, NF-κB p65, TNF-α, and IL-1β, and decreased levels of GSH, SOD, GST, and IL-10, denoting oxidative and inflammatory responses. The necroptosis-related proteins RIP1, RIP3, MLKL, and caspase-8 were upregulated in CIS-treated rats. AZM effectively prevented lung tissue injury, ameliorated oxidative stress and NF-κB p65 and pro-inflammatory markers levels, boosted antioxidants and IL-10, and downregulated necroptosis-related proteins in CIS-administered rats. AZM decreased the concentration of Ang II and increased those of Ang (1-7), cytoglobin, PPARγ, SIRT1, Nrf2, and HO-1 in the lungs of CIS-treated rats. In conclusion, AZM attenuated the lung injury provoked by CIS in rats through the suppression of inflammation, oxidative stress, and necroptosis. The protective effect of AZM was associated with the upregulation of Nrf2/HO-1 signaling, cytoglobin, PPARγ, and SIRT1.

## 1. Introduction

**Figure 1:**
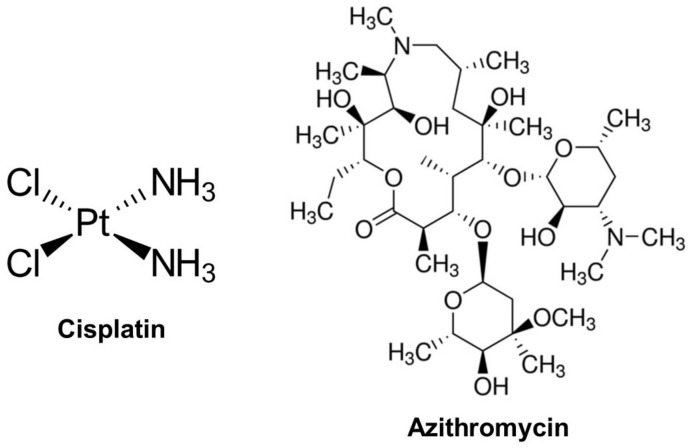
Chemical structure of cisplatin and azithromycin.

Platinum-based chemotherapeutics (platenoids) are commonly used in the treatment of many malignancies, including breast, gastric, lung, and cervical cancers [1,2]. Cisplatin (CIS; Figure 1) is one of the most often utilized platinoids that acts by increasing the generation of free radicals, cross-linking with DNA, and interfering with DNA replication, which results in cell death [2]. The anticancer effectiveness of CIS is dose-dependent, but high doses produce significant detrimental effects on non-cancerous tissues and non-targeted organs [3]. Lung injury is among the serious adverse effects of CIS [4] and the clinical features of its induced acute lung injury (ALI) include non-cardiogenic pulmonary edema and injury to the alveolar-capillary membrane [5]. CIS-associated ALI can culminate in hypoxemia and mortality [5]. The multi-organ toxicity induced by CIS is associated with oxidative stress and inflammation [6,7,8,9]. Inflammation plays a key role in CIS-induced ALI, and a case report published by Ideguchi et al. demonstrated that eosinophilic pneumonia is an adverse effect of CIS [4]. In mice challenged with CIS, inflammatory cell infiltration, thickening of the interalveolar septa, ciliary fragmentation, and elevated levels of reactive oxygen species (ROS) were reported in the lung [10]. In addition to lipid peroxidation (LPO), protein oxidation, and DNA damage, elevated ROS levels can activate nuclear factor-kappaB (NF-κB), which controls the release of inflammatory mediators and cell injury. NF-κB is a transcription factor that is significantly implicated in the inflammatory cascades by promoting the release of downstream pro-inflammatory cytokines, such as TNF-α and IL-1β. These inflammatory mediators increase the inflammatory burden and work in concert with ROS to provoke cell injury via apoptosis [11,12].

Necroptosis is a regulated form of necrosis that follows apoptosis and eventually results in a similar cellular morphology [13]. Necroptosis is implicated in pathological cell death in myocardial infarction, chemotherapy-induced cell death, ischemic brain injury, and other diseases [13]. Necroptosis is linked to the emergence of lung injury in various circumstances, and recent studies have provided substantial evidence for the significance of necroptosis in lung disorders [14,15]. Necroptosis is dependent on the serine-threonine kinase receptor-interacting protein 1 (RIP1) which functions in concert with RIP3. TNF-α, IL-1β, and lysosomal membrane permeabilization (LMP) are among the necroptosis-inducing factors, and RIP3 is required for TNF-α-induced necroptosis [16,17]. Upon activation, the RIP1/RIP3 complex recruits MLKL, resulting in plasma membrane rupture and the release of endogenous molecules [18]. Alveolar type II cells may also contribute to the secretion of growth factors and pro-inflammatory molecules after damage [19]. Oxidative stress is associated with LMP and consequent necrosis [20], and the release of endogenous molecules from the dead cells can trigger an inflammatory response [21,22]. Given the role of oxidative injury and inflammation in the induction of cell death, the attenuation of these pathological processes can protect against CIS-induced ALI.

The activation of several cytoprotective factors can protect against CIS ALI by mitigating oxidative and inflammatory damage. Nuclear factor erythroid 2-related factor 2 (Nrf2) is effective in preventing tissue injury by promoting the transcription of several cytodefensive factors, including heme oxygenase-1 (HO-1) [23]. Upon exposure to ROS and/or electrophiles, Nrf2 dissociates from Keap1, and its nuclear translocation and subsequent binding to ARE promote the transcription of its target genes. Nrf2/HO-1 signaling activation resulted in attenuating the severity of lung injury in different disorders (reviewed in [24]). Sirtuin 1 (SIRT1) is a class III histone deacetylase involved in mitochondrial biogenesis, substrate metabolism, inflammation, and other different cellular processes [25]. The activation of SIRT1 under stress conditions leads to cell survival by regulating several signaling mechanisms [26]. SIRT1 protected the kidney against CIS-induced injury by deacetylating NF-κB and p53 and inhibiting inflammation [27] and ameliorated ALI in lipopolysaccharide (LPS)-challenged mice by decreasing endothelial tight junction permeability and suppressing inflammation [28]. Peroxisome proliferator-activated receptor gamma (PPARγ) is a ligand-activated transcription factor with a predominant role in adipogenesis, which controls genes that are involved in cell differentiation and metabolism. PPARγ activation can confer protection against oxidative stress and the inflammatory response in respiratory diseases [29]. PPARγ can inhibit NF-κB via direct and indirect mechanisms [30], and its upregulation mitigated chemotherapy-induced oxidative damage and the inflammatory response in rodent models [31,32,33].

Azithromycin (AZM; Figure 1) is a macrolide antibiotic used widely to treat respiratory tract infections [34]. AZM has the potential of preventing the inflammatory response characterized by neutrophil influx, cytokine storm, and hypercoagulability by inhibiting NF-κB, which could be of interest in the treatment of COVID-19 [35]. It is one of the safest antibiotics, and its clinical use for respiratory diseases has resulted in few short-term side effects [36,37]. In a trial for COVID-19, AZM was more clinically effective and safer than hydroxychloroquine, and only the patients who received AZM displayed signs of recovery [38]. Headache, dizziness, and gastrointestinal upset were the side effect reported in 1–5% of the patients who received AZM. The use of AZM could be associated with adverse effects in patients with a prolonged QT interval, and cardiovascular deaths were suggested to increase following a 5-day treatment course [39]. In contrast, long-term AZM treatment without occurrences of cardiovascular death was reported in cystic fibrosis patients [40], and AZM intravenous administration in dogs with chronic atrioventricular block did not prolong the QT interval [41]. AZM may enhance the activity of anticancer drugs such as tyrosine kinase or proteasome inhibitors [42] and DNA-damaging drugs [43]. Despite the beneficial effects of AZM, its impact on CIS-induced lung damage has not been studied yet. This investigation aimed to evaluate the potential of AZM to prevent CIS-induced oxidative stress, inflammation, necroptosis, and ALI in rats, pinpointing the involvement of SIRT1, PPARγ, and Nrf2/HO-1 signaling.

## 2. Results

### 2.1. AZM Mitigates CIS-Induced Lung Injury

**Figure 2:**
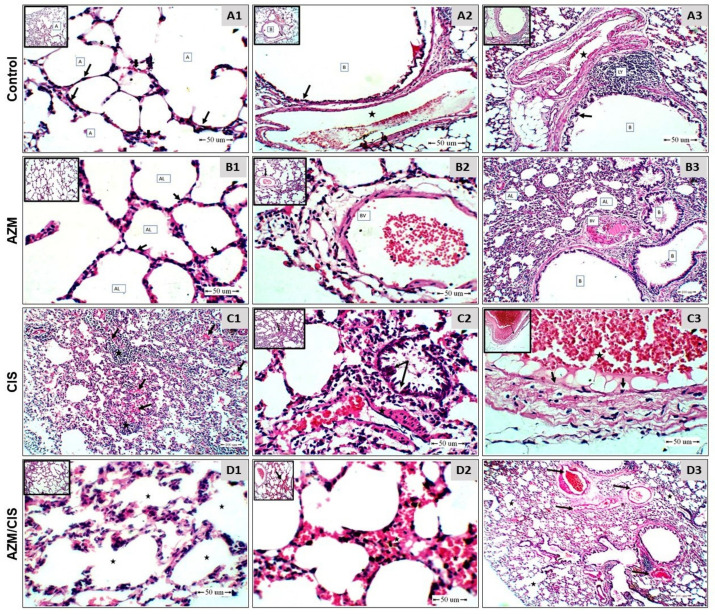
Photomicrographs of sections of the lung of (**A1**–**A3**) control rats showing normal alveoli (A) lined with a flat epithelium (arrow) and separated by septa made of connective tissue with capillaries (arrow) (**A1**), bronchi (**B**) lined with ciliated columnar cells (black arrow), peri-bronchial blood vessels (star), and a peri-bronchial-associated lymphoid follicle (LY) (**A2**,**A3**). (**B1**–**B3**) AZM-treated rats showing normal alveoli (AL), bronchi (B), blood vessels (BV), and interstitial capillaries (arrow). (**C1**–**C3**) CIS-treated rats showing wide-spread pulmonary alveolar edema (black arrow) and a heavy inflammatory cellular reaction (star) (**C1**), damaged bronchiolar epithelium (arrow), a thickened-wall peri-bronchial blood vessel (star) (**C2**), a red thrombus (black star), and damaged endothelium (black arrow) (**C3**). (**D1**–**D3**) CIS-administered rats treated with AZM showing peri-bronchial blood vessels (black arrow) (**D1**) and no evidence of alveolar edema or inflammatory cellular reaction (star) (**D2**,**D3**). (H&E—Scale bar = 50 µm).

Examination of H&E-stained sections of the lung of control and AZM-supplemented rats showed normal alveoli lined with a flat epithelium and separated by septa made of connective tissue that contains capillaries, normal bronchi lined with short ciliated columnar cells, peri-bronchial blood vessel with a thin wall and a wide lumen, and peri-bronchial-associated lymphoid follicles (Figure 2A1–A3,B1–B3). CIS caused multiple alterations, including wide-spread pulmonary edema in the alveoli, a heavy inflammatory cellular reaction in the alveoli and around small blood vessels (Figure 2C1), damage of the bronchiolar epithelium, thickened-wall peri-bronchial blood vessels (Figure 2C2), and red thrombi attached to the damaged endothelium (Figure 2C3). The alveoli of CIS-administered rats treated with AZM showed no evidence of edema or inflammatory cellular reaction, and mild interstitial hemorrhage (Figure 2D1–D3).

### 2.2. AZM Attenuates CIS-Induced Oxidative Stress

**Figure 3:**
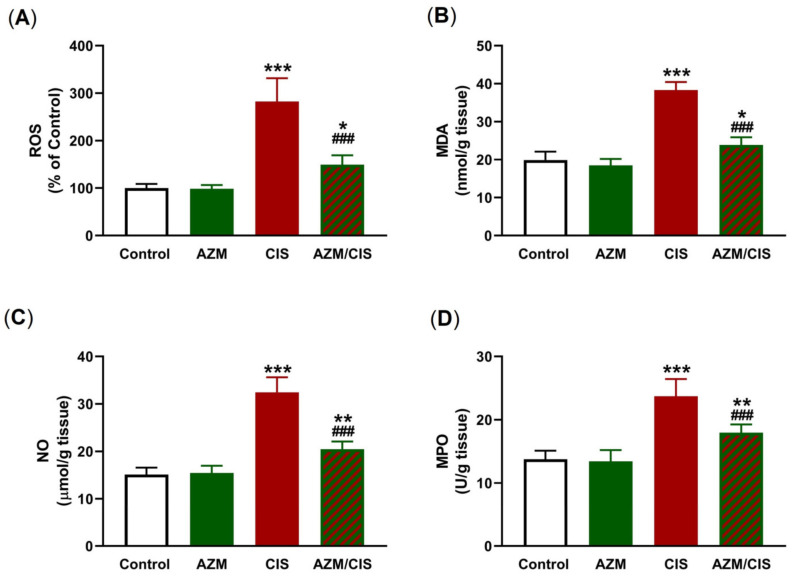
AZM attenuates CIS-induced oxidative stress. AZM decreased the levels of ROS (**A**), MDA (**B**), and NO (**C**), and MPO activity (**D**) in CIS-treated rats. Data are mean ± SEM, (*n* = 8). * *p* < 0.05, ** *p* < 0.01 and *** *p* < 0.001 versus Control and ^###^ *p* < 0.001 versus CIS.

**Figure 4:**
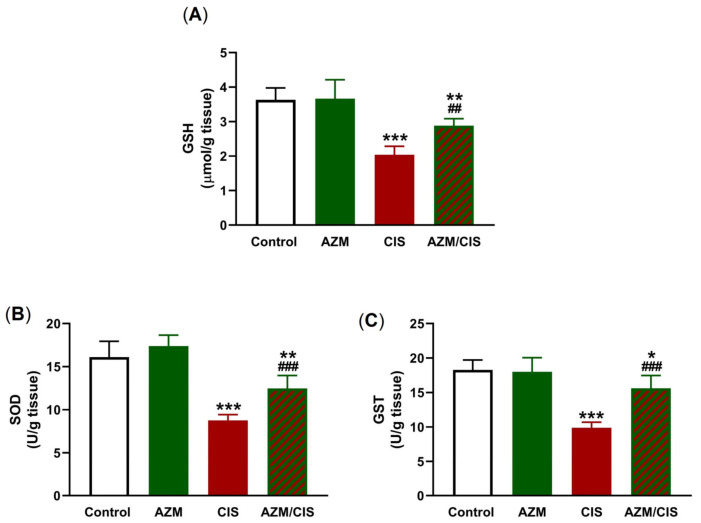
AZM increased the levels of GSH (**A**), and activities of SOD (**B**), and GST (**C**) in CIS-treated rats. Data are mean ± SEM, (*n* = 8). * *p* < 0.05, ** *p* < 0.01 and *** *p* < 0.001 versus Control. ^##^ *p* < 0.01 and ^###^ *p* < 0.001 versus CIS.

The protective effect of AZM against oxidative stress in the lung of CIS-administered rats was assessed by measuring the levels of ROS, malondialdehyde (MDA), nitric oxide (NO), myeloperoxidase (MPO), and antioxidants (Figure 3 and Figure 4). CIS injection resulted in elevated levels of ROS, MDA, NO, and MPO in the lungs of the rats (*p* < 0.001) (Figure 3A–D). The levels of glutathione (GSH), superoxide dismutase (SOD), and glutathione-S-transferase (GST) were decreased in the lungs of CIS-treated rats (Figure 4A–C). AZM effectively decreased MDA, NO, and MPO concentrations and enhanced those of GSH, SOD, and GST in CIS-administered rats, while had no effects on redox homeostasis was evident in normal rats.

### 2.3. AZM Prevents CIS-Induced Inflammation

**Figure 5:**
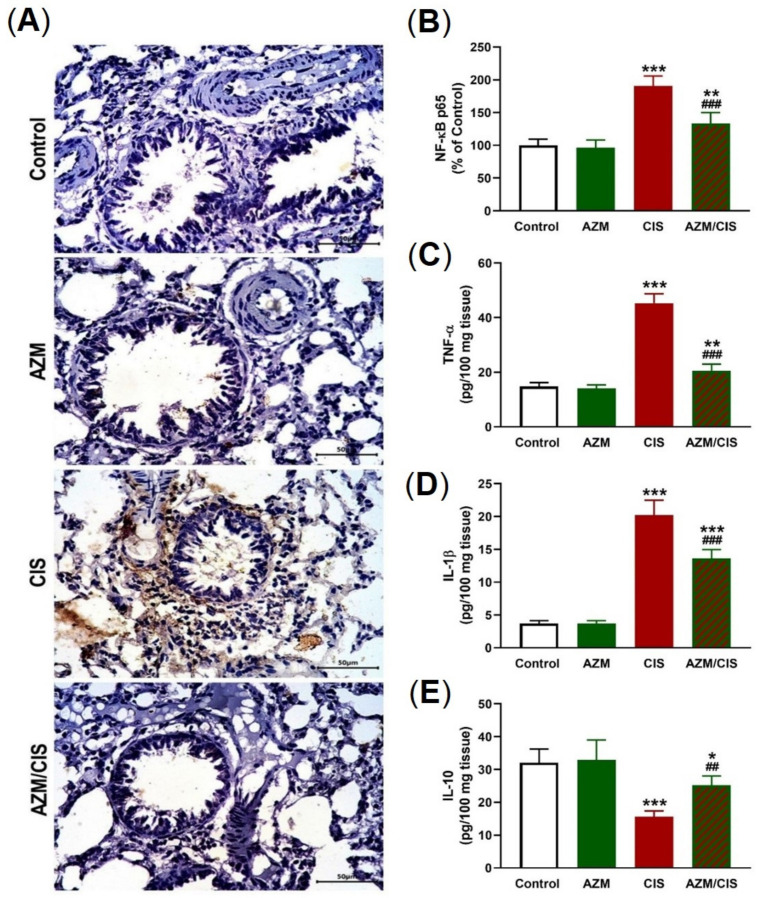
AZM prevented inflammation in CIS-treated rats. (**A**) Photomicrographs showing increased NF-κB p65 in the lung of rats that received CIS and its decrease in AZM-treated rats. (**B**) Image analysis of NF-κB p65 immunostaining. (**C**–**E**) AZM decreased TNF-α (**C**) and IL-1β (**D**), and increased IL-10 (**E**) levels in CIS-treated rats. Data are mean ± SEM, (*n* = 8). * *p* < 0.05, ** *p* < 0.01 and *** *p* < 0.001 versus Control. ^##^ *p* < 0.01 and ^###^ *p* < 0.001 versus CIS.

Immunohistochemical (IHC) staining of NF-κB p65 revealed a significant upregulation in the lungs of CIS-treated rats (*p* < 0.001; Figure 5A,B). TNF-α (Figure 5C) and IL-1β (Figure 5D) levels were elevated, whereas IL-10 (Figure 5E) was downregulated in the lung of CIS-administered rats. AZM downregulated NF-κB p65, TNF-α, and IL-1β and increased the level of IL-10 significantly in CIS-administered rats.

### 2.4. AZM Prevents Necroptosis in the Lungs of CIS-Treated Rats

**Figure 6:**
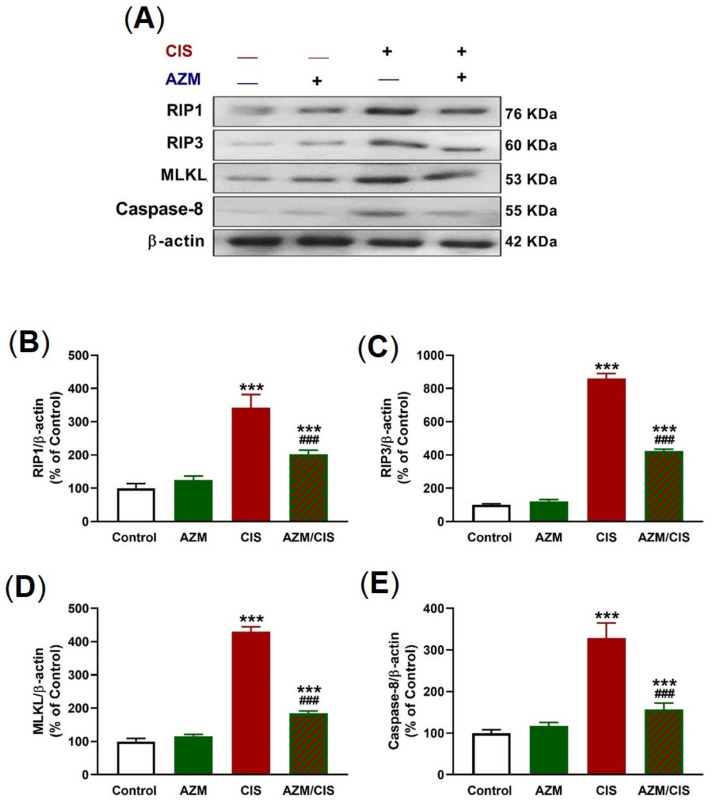
AZM prevented CIS-induced lung necroptosis. (**A**) Representative blots of RIP1, RIP3, MLKL, caspase-8, and β-actin. (**B**–**E**) AZM decreased RIP1 (**B**), RIP3 (**C**), MLKL (**D**), and caspase-8 (**E**) levels in CIS-treated rats. Data are mean ± SEM, (*n* = 8). *** *p* < 0.001 versus Control and ^###^ *p* < 0.001 versus CIS.

The protein levels of RIP1, RIP3, MLKL, and caspase-8 were determined using western blotting (Figure 6A–E) to assess the role of CIS in provoking necroptosis and the protective effect of AZM. CIS increased all assayed necroptosis-related proteins in the lung of rats, an effect that was reversed in AZM-treated rats.

### 2.5. AZM Upregulates Nrf2/HO-1 Signaling, SIRT1, PPARγ, and Cytoglobin (Cygb) in CIS-Treated Rats

**Figure 7:**
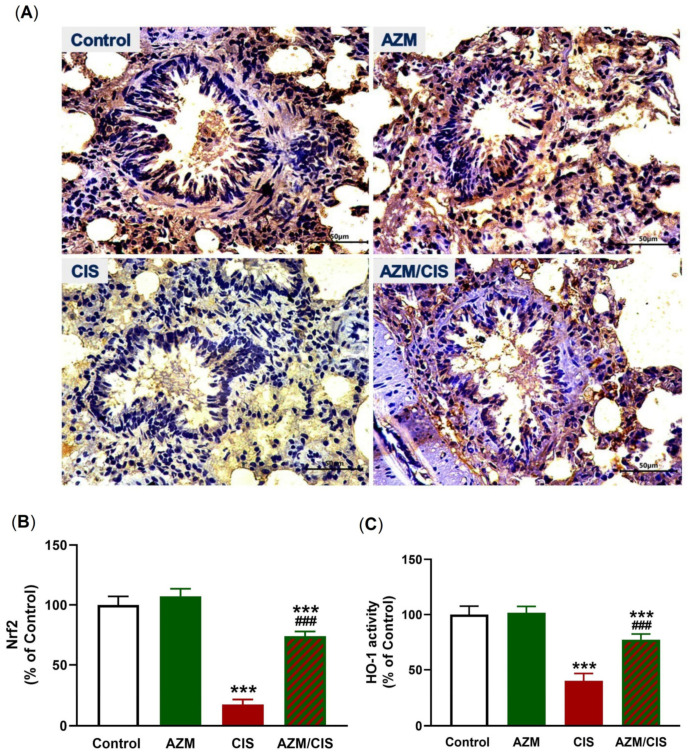
AZM upregulated Nrf2/HO-1 in the lung of CIS-treated rats. (**A**) Photomicrographs and (**B**) image analysis revealing increased Nrf2 and (**C**) increased HO-1 activity in the lung of CIS-administered rats treated with AZM. Data are mean ± SEM, (*n* = 8). *** *p* < 0.001 versus Control and ^###^ *p* < 0.001 versus CIS.

**Figure 8:**
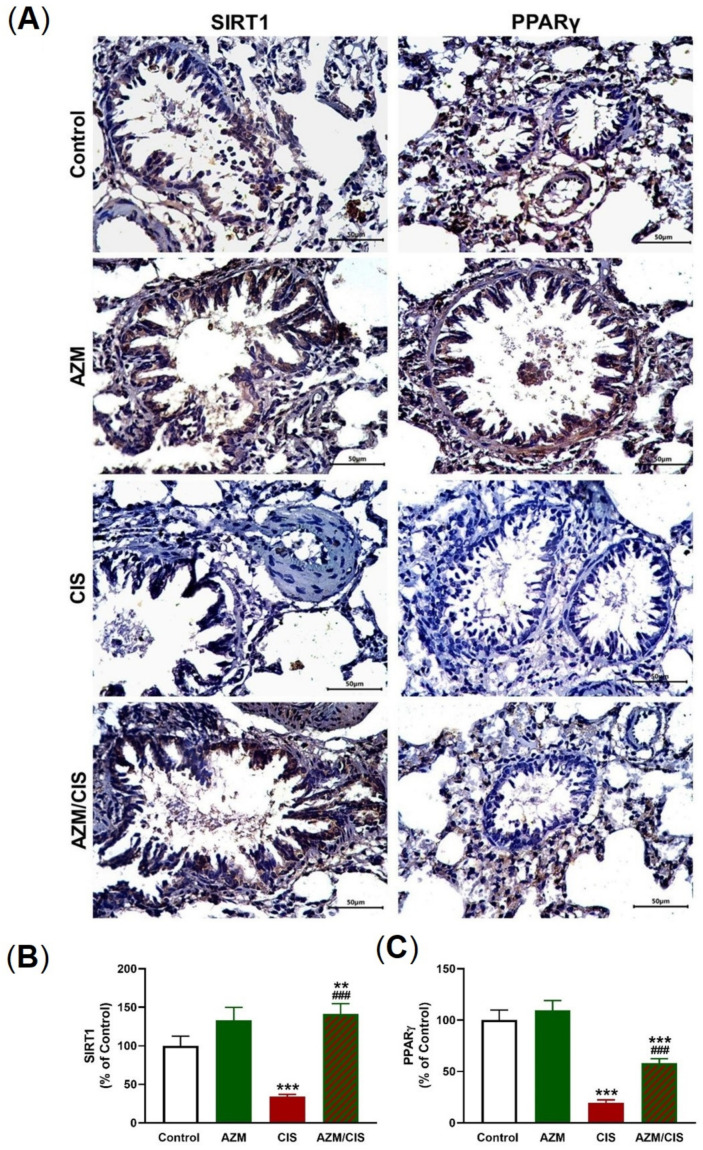
AZM upregulated lung SIRT1 and PPARγ in CIS-treated rats. CIS decreased SIRT1 (**A**,**B**) and PPARγ (**A**,**C**) levels, effects that were prevented in AZM-treated rats. Data are mean ± SEM, (*n* = 8). ** *p* < 0.01 and *** *p* < 0.001 versus Control and ^###^ *p* < 0.001 versus CIS.

**Figure 9:**
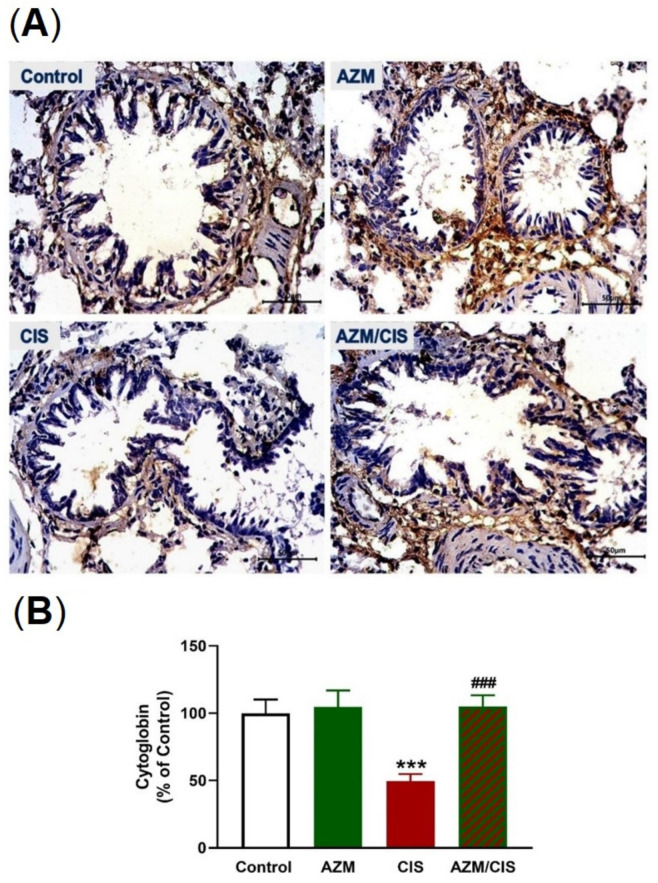
AZM increased cytoglobin in the lungs of CIS-treated rats. (**A**) Photomicrographs and (**B**) image analysis revealing increased cytoglobin level the lungs of CIS-administered rats treated with AZM. Data are mean ± SEM, (*n* = 8). *** *p* < 0.001 versus Control and ^###^ *p* < 0.001 versus CIS.

As presented in Figure 7A–C, CIS downregulated Nrf2 and HO-1 in the lung of rats (*p* < 0.001). Figure 8A–C showed that CIS decreased SIRT1 and PPARγ levels significantly in the lung of rats (*p* < 0.001). Similarly, the data in Figure 9A,B showed a significant downregulation of Cygb in the lungs of CIS-treated rats. AZM prevented the negative impact of CIS on SIRT1, PPARγ, and Cygb in the lung of rats.

### 2.6. AZM Decreases Ang II and Increases Ang (1-7) in CIS-Administered Rats

**Figure 10:**
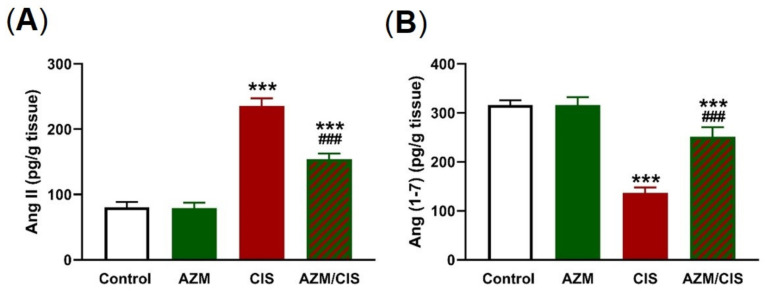
AZM decreased Ang II (**A**) and increased Ang (1-7) levels (**B**) in the lungs of CIS-treated rats. Data are mean ± SEM, (*n* = 8). *** *p* < 0.001 versus Control and ^###^ *p* < 0.001 versus CIS.

CIS administration resulted in a remarkable elevation of Ang II levels (Figure 10A) and decreased Ang (1-7) levels (Figure 10B) in the lungs of rats (*p* < 0.001). Treatment with AZM ameliorated Ang II and Ang (1-7) levels in CIS-administered rats, with no effect in normal rats.

## 3. Discussion

Cisplatin (CIS) is used widely to treat several malignancies; however, its adverse effects limit its therapeutic application [3]. ALI is a serious side effect of CIS, with eosinophilic pneumonia, non-cardiogenic pulmonary edema, and alveolar-capillary membrane injury representing the characteristic clinical features [4,5]. Oxidative stress and inflammation-mediated tissue injury are implicated in CIS-induced cell death and lung injury [9,10,44]. Thus, attenuation of these pathological processes might be effective against CIS-induced ALI. Given the immunomodulatory potential and the beneficial effect of AZM in preventing the inflammatory response [35], we evaluated its protective role against ALI, with emphasis on oxidative stress, inflammation, necroptosis, and the possible involvement of SIRT1 and PPARγ.

The administration of CIS resulted in tissue injury in the lungs of rats, characterized by wide-spread pulmonary edema, a heavy inflammatory reaction in the alveoli and around small blood vessels, damaged bronchiolar epithelium, thick-walled peri-bronchial blood vessels, and red thrombi attached to the damaged endothelium. Very recently, we demonstrated venous congestion, inflammatory exudates, thickened interalveolar septa and arterial wall, and perivascular edema in the lung of CIS-administered rats [44]. In support to these findings, infiltrating inflammatory cells, alveolar edema and damage, hemorrhage, and fibrotic changes were reported in other studies [8,9]. AZM effectively prevented CIS-induced lung tissue injury, with no evidence of edema or inflammatory cellular reaction in the alveoli, pinpointing its potent protective effect.

The generation of ROS plays a central role in the cytotoxic and anti-cancer activities of CIS [2]. This property is associated with adverse effects mediated via oxidative stress, inflammation, and cell death in normal tissues. In this context, CIS increased the levels of ROS in mice [10] and decreased those of antioxidants in the rat lung [44]. Excess ROS can damage cell membrane lipids, proteins, DNA, and other cellular macromolecules via their versatile prooxidant activity. Consequently, both membrane fluidity and permeability are disrupted, and antioxidant enzymes and GSH are depleted, leading to cell death [45]. In this study, CIS increased ROS and MDA levels and decreased GSH, SOD, and GST levels, denoting an oxidative stress status. These findings are in accordance with previous work from our lab [44] as well as others [46], showing elevated MDA concentrations along with declined levels of GSH and antioxidant enzymes. Additionally, NO and MPO activity were increased in the lung of CIS-treated rats. NO is generated via iNOS activation and form peroxynitrite by a reaction with superoxide, and the product oxidizes DNA and increases the ROS levels [47]. MPO, an enzyme produced by neutrophils, is implicated in different lung pathologies in which inflammation is central [48], and a recent study showed its elevation in the lungs of CIS-administered rats [44]. The activation of MPO leads to the formation of pro-oxidant species, resulting in oxidation, nitration, halogenation, or cross-linking of proteins [49]. Besides the damaging oxidative consequences, ROS provoke inflammation by activating NF-κB and the release of downstream pro-inflammatory cytokines [11,12]. CIS-administered rats in this study exhibited inflammation, marked by upregulated NF-κB p65, TNF-α, and IL-1β, decreased IL-10, and the presence of an inflammatory reaction in the alveoli and around small blood vessels. CIS can promote NF-κB and the development of an inflammatory response through the ROS-mediated activation of TLR-4 signaling, as we previously reported [44]. The activation of TLR-4 and its subsequent inflammatory responses in response to microbial and non-microbial stimuli were linked to ALI [50].

Both oxidative and inflammatory responses provoked by CIS in the lungs of rats were prevented by AZM. Treatment with AZM decreased ROS, MDA, NO, MPO, NF-κB p65, TNF-α, and IL-1β levels, and upregulated GSH, SOD, GST, and IL-10 levels. These findings support the notion that AZM can mitigate CIS-induced ALI by attenuating oxidative stress and inflammation. Accordingly, AZM was the most effective drug among others that protected the stomach against alcohol-induced damage by decreasing LPO and boosting the activities of many antioxidant enzymes [51]. In human alveolar cells, AZM prevented cigarette smoke extract-mediated ROS generation and cell death [52]. The protective effect of AZM against cigarette smoke extract-induced damage in alveolar epithelial cells was explained by the activation of Nrf2 [53], a redox-sensitive factor that promotes the transcription of multiple antioxidant and cytodefensive factors [23]. The efficacy of AZM against inflammation was showed in different studies. In murine pulmonary neutrophilia, AZM attenuated inflammation by inhibiting the production of IL-1β by macrophages [54]. In patients with systemic lupus erythematosus, the beneficial anti-inflammatory effect of AZM was explained via its ability to promote an alternatively activated macrophage phenotype [55]. Another study in 50 lung transplant recipients demonstrated acute allograft dysfunction and found that AZM reduced the number of neutrophils in the peripheral blood and inflamed tissues in cystic fibrosis patients, resulting in a clinical improvement in lung allograft function [56]. Moreover, a recent investigation suggested the beneficial effect of AZM in COVID-19 patients due to its suppressive effects on the hyperinflammation caused by cytokine release [57]. AZM prevented NF-κB activation and reduced the production of inflammatory cytokines in tracheal aspirate cells from preterm newborns [58]. It inhibited NF-κB in an acute model of lung inflammation induced by LPS and hence decreased inflammatory cell infiltration into the lung tissue and the release of pro-inflammatory cytokines in the alveolar space [59]. Our findings added further support to the anti-inflammatory activity of AZM by showing its ability to downregulate NF-κB and its controlled pro-inflammatory mediators and to increase IL-10.

Pro-inflammatory mediators and redox imbalance work in concert to promote apoptotic cell death via disrupting the mitochondrial membrane potential (MMP) and the release of cytochrome c, leading to caspase-3 activation and the degradation of cellular proteins and DNA and, consequently, to cell death [60]. We recently reported the upregulation of the pro-apoptotic mediators Bax and caspase-3 in the lungs and kidney of CIS-administered rodents [44,61,62]. Necroptosis, a regulated form of necrosis that follows apoptosis, has been recently linked to lung injury in pulmonary disorders [14,15]. In the present study, CIS provoked necroptosis in the lungs of rats, as shown by the upregulation of RIP1, RIP3, MLKL, and caspase-8. ROS, TNF-α, and IL-1β can activate necroptosis by activating the RIP1/RIP3 complex that recruits MLKL, leading to damage of the plasma membrane and cell death, and the release of endogenous molecules that trigger further inflammation [16,18,20,21]. AZM effectively downregulated RIP1, RIP3, MLKL, and caspase-8 in the lungs of CIS-administered rats, an effect that could be attributed to the suppression of oxidative stress and inflammation.

We assumed that the upregulation of Nrf2/HO-1, PPARγ, SIRT1, and Cygb contributed to the protective efficacy of AZM against CIS-induced ALI. CIS decreased the expression levels of Nrf2, HO-1, PPARγ, SIRT1, and Cygb, an effect that was markedly prevented in AZM-treated rats. AZM upregulated Nrf2 and HO-1 in the lungs of CIS-treated rats, an effect that explained the enhanced cellular antioxidants. Besides the attenuation of oxidative damage, Nrf2 and HO-1 can prevent inflammation by inhibiting NF-κB and activating anti-inflammatory mechanisms [63]. The activation of PPARγ can confer protection against oxidative stress and the inflammatory response in the presence of respiratory diseases [29] and in rodents challenged with chemotherapy [31,32,33]. During allergic airway inflammation, the activation of PPARγ downregulated the release of mucin and pro-inflammatory mediators in bronchial epithelial cells [64]. The anti-inflammatory activity of PPARγ is mediated via the direct and indirect inhibition of NF-κB [30] and the induction of HO-1 [65]. The transcription of HO-1 is directly regulated by PPARγ and, upon activation, can attenuate inflammation, ROS production, and apoptotic cell death [65]. The activation of HO-1 by treating LPS-challenged pulmonary alveolar epithelial cells with PPARγ agonists prevented the inflammatory response by suppressing NF-κB [66]. Besides PPARγ, AZM upregulated SIRT1 in the lungs of CIS-administered rats. SIRT1 can suppress inflammation by deacetylating NF-κB and can prevent CIS-induced kidney injury [27]. It inhibited inflammation and decreased endothelial tight junction permeability in LPS-challenged mice [28]. SIRT1 deacetylation promotes PGC-1α, Nrf1, and Nrf2 activation, resulting in the transcription of mitochondrial transcription factor A (TFAM) and its transfer to the mitochondria and the promotion of mitochondrial biogenesis [67]. In lung epithelial cells, SIRT1 activated PGC-1α and Nrf1 and prevented mitochondrial dysfunction and apoptosis induced by hypoxia [68]. Improved MMP and enhanced cytochrome c oxidase 1 and ATP are among the beneficial effects of SIRT1/PGC-1α/NRF/TFAM signaling activation, which protected against lung oxidative stress and inflammation in murine COPD [69]. Cygb is an intracellular respiratory protein that can scavenge ROS, prevent oxidative stress, and maintain redox homeostasis [70]. Overexpression of Cygb protected against pro-oxidant injury by decreasing chemically induced ROS, whereas its downregulation sensitized the cells to oxidative DNA damage and cell injury [70,71]. Increased susceptibility to radiation-induced injury, fibrosis, and inflammation has been reported in cells and organs lacking Cygb [70]. Recently, Cygb has been shown to exhibit SOD activity, decrease superoxide, NO, and peroxynitrite effectively, and prevent oxidative injury [72].

The renin–angiotensin system (RAS) is involved in cardiovascular, adrenal, renal, and other functions through the effects of Ang-II [73]. It is implicated in inflammatory diseases, and its modulating drugs have shown anti-inflammatory, anti-stress, and anti-apoptosis activities [44,74,75]. Ang II elicits the release of inflammatory mediators from endothelial [76], smooth muscle [77], and renal tubular cells [78]. In contrast, Ang 1-7 counteracts the Ang II/AT1R axis and promotes vasodilation and anti-inflammatory, anti-angiogenic, and antihypertensive effects [79]. In the current study, Ang II was elevated, and Ang 1–7 was decreased in the lung of CIS-administered rats, as we previously reported [44]. These alterations were associated with upregulated NF-κB and increased levels of pro-inflammatory cytokines. Interestingly, AZM decreased the levels of Ang II and increased those of Ang (1-7) in the lungs of CIS-administered rats. Very recently, we demonstrated the protective effect of the Ang II receptor blocker candesartan against CIS-induced oxidative stress, inflammation, and lung injury in rats [44]. Therefore, modulation of the components of the RAS was implicated in the anti-inflammatory, antioxidant, and protective effects of AZM against CIS-induced ALI.

## 4. Materials and Methods

### 4.1. Animals and Treatments

Thirty-two male albino rats, weighing 190–210 g, obtained from the central animal house, Assiut University (Egypt), were included in this investigation. The animals were housed under standard conditions on a 12 h light/dark cycle, with free access to food and water. The animal study protocol was approved by the Research Ethics Committee of Al-Azhar University (ZA-AS/PH/12/C/2022). The rats were allocated into four groups (*n* = 8) as follows:

Group I (Control): received the vehicle (0.5% carboxymethyl cellulose (CMC; (Sigma, St. Louis, MO, USA)) for 10 days.

Group II (AZM): received 25 mg/kg AZM [80] (Sigma, St. Louis, MO, USA) for 10 days.

Group III (CIS): received 0.5% CMC for 10 days and 7 mg/kg CIS (Sigma, St. Louis, MO, USA) [6] on day 7.

Group IV (AZM/CIS): received 25 mg/kg AZM [80] for 10 days and 7 mg/kg CIS [6] on day 7.

AZM and CIS were dissolved in 0.5% CMC and physiological saline and administered vial oral gavage and intraperitoneal (i.p.) injection, respectively. The rats in groups I and II received an i.p. injection of saline on day 7. At the end of the experiment, the rats were anesthetized with ketamine (100 mg/kg i.p.), and blood was collected via cardiac puncture for serum preparation. Following immediate scarification and dissection, the lungs were excised and divided into several sections. One part was fixed in 10% neutral buffered formalin (NBF) for histological and IHC investigations, whereas another part was homogenized (10% *w*/*v*) in Tris-HCl buffer (pH = 7.4) and centrifuged, and the supernatant was separated for biochemical assays. Other parts were homogenized in RIPA buffer with protease inhibitors and centrifuged, and the supernatant was used for Western blotting. Protein concentration in the supernatant was determined using the Bradford reagent [81].

### 4.2. Histopathology and IHC Examination

Lung samples fixed in 10% NBF for 24 h were processed for routine paraffin embedding, and 4 µm sections were cut. The sections were stained with hematoxylin and eosin [82] and examined with a light microscope. For IHC investigation, other sections were deparaffinized, cleared, and treated with 50 mM citrate buffer (pH = 6.8) and then blocked with 1% bovine serum albumin. Following the blocking of endogenous peroxidase using 0.3% hydrogen peroxide (H_2_O_2_), the sections were washed with phosphate-buffered saline (PBS) and probed with primary antibodies against SIRT1, PPARγ, NF-κB p65, and Cygb (Biospes, Chongqing, China) overnight at 4 °C. Following washing and incubation with the secondary antibodies, color development was carried out using DAB in H_2_O_2_, and hematoxylin was employed for counterstaining. The intensity of the obtained color was determined using ImageJ (NIH, Bethesda, MD, USA).

### 4.3. Biochemical Assays 

ROS were assayed by mixing the samples with H_2_DCF-DA (Sigma, St. Louis, MO, USA), followed by incubation at 37  °C for 30 min and measuring the fluorescence at 490 nm [83]. To determine the MDA levels, the samples (0.2 mL), 0.6% thiobarbituric acid (0.4 mL), and 1% O-phosphoric acid (1.2 mL) were mixed and heated for 45 min at 95 °C. n-butanol (0.8 mL) was added, and the mixture was vortexed and centriguged for 10 min at 2000× *g* rpm, and the absorbance of the upper layer was measured at 535 nm [84]. GSH was measured by mixing the samples with Ellman’s reagent and measuring the absorbance of the obtained yellow color at 412 nm [85]. To measure NO levels, equal volumes of the samples and Griess reagent were mixed and incubated for 10 min, and the absorbance was measured at 540 nm. [86]. SOD activity was determined by monitoring the inhibition of pyrogallol autoxidation as described by Marklund and Marklund [87]. GST activity was measured by mixing the samples with 0.1 M phosphate buffer (pH 6.5), GSH, and 2,4-dinitrochlorobenzene, and monitoring the absorbance change for 3 min at 340 nm [88]. HO-1 activity was assayed by mixing the samples with glucose-6-phosphate (2 mM), NADPH (0.8 mM), hemin (20 µM), and glucose-6-phosphate dehydrogenase (0.2 U). The mixture was incubated for 1 h at 37 °C, and the absorbance was measured at 464 nm [89]. The activity of MPO was determined by mixing the samples with 50 mM phosphate buffer, o-dianisidine hydrochloride, and H_2_O_2_. The changes in the absorbance were measured spectrophotometrically at 460 nm [90]. All used chemicals were supplied by Sigma (St. Louis, MO, USA). ELISA kits purchased from ELabscience (Wuhan, China) were used to determine the levels of TNF-α, IL-1β, IL-10, Ang-II, and Ang-(1-7) according to the provided instructions.

### 4.4. Western Blotting

To evaluate the effect of CIS and AZM on RIP1, RIP3, MLKL, and caspase-8, 50 µg protein was subjected to SDS-PAGE followed by transfer onto PVDF membranes. Then, 5% BSA was used for blocking, and the membranes were then incubated with anti-RIP1 (sc-133102—Santa Cruz, Dallas, TX, USA), anti-RIP3 (sc-374639—Santa Cruz, Dallas, TX, USA), anti-MLKL (YPA2507—Biospes, Chongqing, China), anti-caspase-8 (sc-70501—Santa Cruz, USA), and anti-β-actin (sc-8432—Santa Cruz, Dallas, TX, USA) overnight at 4 °C. After washing in TBST, the membranes were incubated with the secondary antibodies for 1 h at RT, washed and visualized using the BCIP/NBT substrate detection kit (GeneMed Biotechnologies, San Francisco, CA, USA). The intensity of the developed bands was determined using ImageJ (NIH, Bethesda, MD, USA).

### 4.5. Statistical Analysis

The results are expressed as mean ± SEM, and one-way ANOVA followed by Tukey’s test was employed to compare the differences among the groups. The statistical analysis was conducted using GraphPad Prism 8.0, and a *p* < 0.05 was considered statistically significant.

## 5. Conclusions

The results of this study provide new information on the protective effect of AZM against CIS-induced ALI. AZM prevented bronchiolar and alveolar injury, suppressed ROS, LPO, NO, MPO, NF-κB and pro-inflammatory mediators, and boosted the antioxidant defenses in CIS-administered rats. This study showed the involvement of necroptosis in CIS-induced ALI and the protective effect of AZM that downregulated RIP1, RIP3, MLKL, and caspase-8. The protective effect of AZM was associated with the upregulation of Nrf2/HO-1 signaling, PPARγ, Cygb, and SIRT1. Thus, in addition to its antibiotic efficacy, AZM can protect the lungs against the toxicity of CIS, and its use in clinical settings could be an effective approach to prevent pulmonary drug toxicity. 

## Data Availability

Data are contained within the article.
